# Legionnaires’ Disease and Travel in Europe

**DOI:** 10.3201/eid1210.060525

**Published:** 2006-10

**Authors:** Rosa Cano, Sophie Jarraud, Joana Pardos, Christine Campese, Carmen Pelaz

**Affiliations:** * Instituto de Salud Carlos III, Madrid, Spain;; † Institut National de la Santé et de la Recherche Médicale E0230, Lyon, France;; ‡ Public Health Laboratory of Girona, Catalonia, Spain;; § Institut de Veille Sanitaire, Saint-Maurice, France

**Keywords:** Outbreak, legionellosis, Europe, EWGLINET, sequence-based typing, letter

**To the Editor:** The European Working Group for *Legionella* Infections (EWGLINET) conducts epidemiologic surveillance of Legionnaires' disease cases associated with travel (*1*) and provides epidemiologic typing markers of *Legionella pneumophila* among reference laboratories in collaborating countries. The procedures and criteria of notification are found in the Guidelines for Control and Prevention of Travel Associated Legionnaires' Disease (*2*). However, establishing the association of >1 case of this disease and a specific tourist accommodation site is difficult because of low attack rates and dispersal of people from the source of infection during the incubation period.

Collaboration promoted by this working group encourages the exchange of data instead of cultures. This distinction is critical when research is conducted on travel-associated Legionnaires' disease, in which strains from patients and environmental sources of infection studied are in different laboratories.

The value of such information is shown in a complex case study that was recently investigated. During July and August 2005, two patients with Legionnaire's disease living in 2 countries in Europe were reported to EWGLINET. Patient 1 was a 45-year-old woman who traveled in France and Spain July 1–6, 2005. Her symptoms started on July 6, 2005, when she was in Girona, Spain, where she was hospitalized. Patient 2 was a 56-year-old woman who traveled in Spain and France August 16–21, 2005. Her symptoms started on August 8, 2005, when she was in France, where she was hospitalized. Both patients tested positive for *L*. *pneumophila* serogroup 1 by specific urinary antigen test and culture, but they recovered and were discharged.

After routine notification to EWGLINET, it was established from the list of accommodation sites provided by the 2 patients that they each had stayed for 1 night at the same hotel in a French city within a 45-day interval. This finding led us to identify a cluster according to the definition in use (2 cases associated with the same accommodation within 2 years) (*2*). However, patient 2 spent 1 day in August in Zaragoza, Spain, during which an outbreak of Legionnaires' disease in the city affected 30 persons. Thus, illness in patient 2 could have been associated with the Zaragoza outbreak. Alternatively, both patients could have contracted the illness independently at different sites. Before onset, patient 1 stayed 5 days in her private residence in Girona and patient 2 visited 3 other hotels.

As soon as cultures from the 2 patients were available, the National Reference Laboratories of France and Spain shared their respective microbiologic results. Since both strains were identified as *L*. *pneumophila* serogroup 1, we performed sequence-based typing (SBT) (*3*) of 6 genes (*fla*A, *pil*E, *asd*, *mip*, *momp*S, and *pro*A) by using the protocol and database of EWGLINET. Both isolates showed identical SBT patterns (2,3,18,15,2,1).

Isolates from 4 patients in the Zaragoza outbreak were identified at the Spanish Reference Laboratory as *L*. *pneumophila* serogroup 1 (Philadelphia monoclonal antibody type) and had identical SBT patterns (3,4,1,1,14,9). Collaboration between public health authorities in France and Spain enabled us to eliminate the association of patient 2 with the Zaragoza outbreak and establish an association of both patients with the same site in France. Control measures were taken at the hotel, but we could not obtain environmental cultures for comparison with those of the patients. Lack of environmental data prevented investigation of the relationship with the other accommodation sites visited.

The SBT method provides robust genotyping with high discriminatory power (index of discrimination >0.94) (*3*). This method is less effective at discriminating between strains than pulsed-field gel electrophoresis (*4*), but it shows excellent reproducibility and may be useful in epidemiologic investigation of outbreaks caused by *L*. *pneumophila*. The availability of an online database with accessible information is key for sharing results and determining the geographic distribution of isolates that cause Legionnaires' disease (*4**,**5*).

This study demonstrates the critical role of sharing results between countries that participate in a network. Agreement is essential on a standardized questionnaire that includes more information on the patient's exposure to a disease. Moreover, despite the performance of the urine antigen test, cultures of clinical samples should be encouraged by clinicians and microbiologists. This step would permit use of techniques, such as SBT, in reference laboratories and sharing of results. Our investigation would have been more difficult without this technique in identifying the site where the infection potentially originated.
